# Design and application of a CA-BDI model to determine farmers’ land-use behavior

**DOI:** 10.1186/s40064-016-3245-7

**Published:** 2016-09-15

**Authors:** Xiaoying Liang, Hai Chen, Yanni Wang, Shixiong Song

**Affiliations:** College of Urban and Environmental Science, Northwest University, Xi’an, Shaanxi China

**Keywords:** CA-BDI, Farmers, Land-use decision-making, Simulation

## Abstract

The belief-desire-intention (BDI) model has been widely used to construct reasoning systems for complex tasks in dynamic environments. We have designed a capabilities and abilities (CA)-BDI farmer decision-making model, which is an extension of the BDI architecture and includes internal representations for farmer household Capabilities and Abilities. This model is used to explore farmer learning mechanisms and to simulate the bounded rational decisions made by farmer households. Our case study focuses on the Gaoqu Commune of Mizhi County, Shaanxi Province, China, where scallion is one of the main cash crops. After comparing the differences between actual land-use changes from 2007 to 2009 and the simulation results, we analyze the validity of the model and discuss the potential and limitations of the farmer land-use decision-making model under three scenarios. Based on the design and implementation of the model, the following conclusions can be drawn: (1) the CA-BDI framework is an appropriate model for exploring learning mechanisms and simulating bounded rational decisions; and (2) local governments should encourage scallion planting by assisting scallion farmer cooperatives and farmers to understand the market risk, standardize the rules of their cooperation, and supervise the contracts made between scallion cooperatives and farmers.

## Background

Analyzing the interactions between environmental or agricultural policies and farmer behavior is generally considered crucial for the sustainability of agro-ecosystems (Evrendilek and Doygun 2000; Parker et al. 2003; Fischbacher et al. 2001; Rammel et al. 2007). A significant amount of recent research has focused on farmer land-use behavior and its impact on agricultural land-use change (Evrendilek and Doygun 2000; Brown et al. 2013). However, the methods by which changes in agricultural policies affect farmer land-use behavior are not well understood (Thompson and Scoones 2009). Future research should focus on identifying the methods by which agricultural policies affect farmer land-use behavior and determine how changes in such behavior influence agricultural land-use changes (Manson 2001; Feola and Binder 2010). The core of the above question is to use an explicit and well-motivated behavioral theory to investigate agents’ behavior and its relationship with system dynamics (Parker et al. 2003; Janssen and Ostrom 2006; Matthews and Selman 2006).

As one of the most popular models of agent decision making (Georgeff et al. 1999), the belief-desire-intention (BDI) model has been widely used to construct reasoning systems for complex tasks in dynamic environments (BoSS et al. 2010). There exist two major criticisms of traditional BDI: the one is that it assumes agents to behave in line with rationality (Wooldridge 2000); the other is that it does not provide any specification of agent communication, which it facilitated learning (Phung et al. 2005). Many extensions have been developed to overcome the restrictions of the original BDI model: although the emotional BDI model (eBDI) did not consider learning and social relations (Moridis and Economides 2008), eBDI gave a new way to address rational agent criticism, and Normative agent architecture (Castelfranchi 1999) and EMIL-A agent architecture (Savarimuthu et al. 2010) reveal the process that agents undergo to learn the norms in a society (Balke and Gilbert 2014). However, emotions or other affective elements are not included in these models. At present, there are few researches using BDI model to construct agents’ land use behavior to simulate agriculture/urban land use change. However, these researches adopt maximum benefit theory to reveal the change of the agent’s behavior (Chen et al. 2009; Ligtenberg and Wachowicz 2004). Therefore, it is necessary to adopt the bounded rational decision-making theory and incorporate communication among agents into human decision-making processes.

The specific aims of this paper are to (1) construct an appropriate farmer decision-making model to explore the bounded rational decisions made by farmers, (2) reveal the mechanism of communication through interactions among different farmer groups and different polices, and (3) simulate changes in farmer land-use behavior and the effect of such changes on agricultural land-use.

The paper provides a brief introduction of the case study area, followed by a review of the primary data collection methods and sample design. Next, a farmer decision-making model is developed to integrate the strengths of the two previously mentioned aspects in explaining human behavior. The results from the model simulation are compared with the actual observed land use, and the process we used to validate the model is described. Possible land-use change in the study area, which is projected to 2015, is discussed under three local measure scenarios. Finally, recommendations based on the main findings of the study are provided in the conclusion and discussion.

## Materials and Methods

### Study area

To mitigate environmental deterioration and foster regional socio-economic development in western China, the Chinese government began implementing a national project known as “Grain for Green” (GFG) in 1999. GFG promotes the conversion of cropland to natural land cover (such as forests and grassland) through a set of cash and in-kind payments to farmers for each hectare enrolled. The goals of the program are to foster regional socio-economic development and reduce the damages caused by cultivation on steep hillside slopes. For example, agriculture-related erosion and degradation within the catchments areas of rivers such as the Yellow and the Yangtze reduce the sustainability of local farmers’ livelihoods and have caused devastating downstream flooding events (Gao et al. 2006). This project has been extended to 2015 (State Council of China 2007). By 2006, 26.9 million hectares of cropland located on steep slopes with a gradient of 25° or greater were returned to forest or grassland in western China. At this time, it was estimated that more than 32 million farmers across 25 provinces had participated in the GFG project (Ye and Fang 2012). Maintaining GFG achievements in western China is predicated on the ability of the program to improve the ecological environment while simultaneously promoting economic development.

Mizhi County, Shaanxi Province (109°49′–110°29′E, 37°39′–38°5′N) is a hilly area of the Loess Plateau, and it was selected as the area for this study. The land area of the county is 1212 km^2^. Prior to the enforcement of the GFG project in 1999, 365 km^2^ of land was under severe soil erosion condition. To halt the severe soil erosion of the catchment of the Yellow River and rehabilitate the ecological functions of the region, the State Forestry Administration of China initiated the GFG project in 1999 in 174 counties nationwide (out of a total of 2861 counties). Mizhi was one of the selected counties. The study of Mizhi County may provide a good reference for the effects of the GFG policy on farmer household land-use behavior and subsequent land-use changes, and the results may inform policies aimed at adjusting land-use behavior in similar areas.

In the first stage (1999–2006) of the GFG project, 115.7 km^2^ of cropland in Mizhi had been returned to forest or grassland. The coverage rate of forest and grassland increased from 31.3 % in 1999 to 40.8 % in 2006. Situated in the north of Mizhi, the Gaoqu Commune experienced the greatest changes in woodland and cropland areas at annual rates of 3.3 and −3.2 %, respectively. The present study focuses on the Gaoqu Commune of Mizhi County to detail how the GFG project and local measures have influenced the land-use behavior of different farmer households and subsequent land-use changes.

**Fig. 1:**
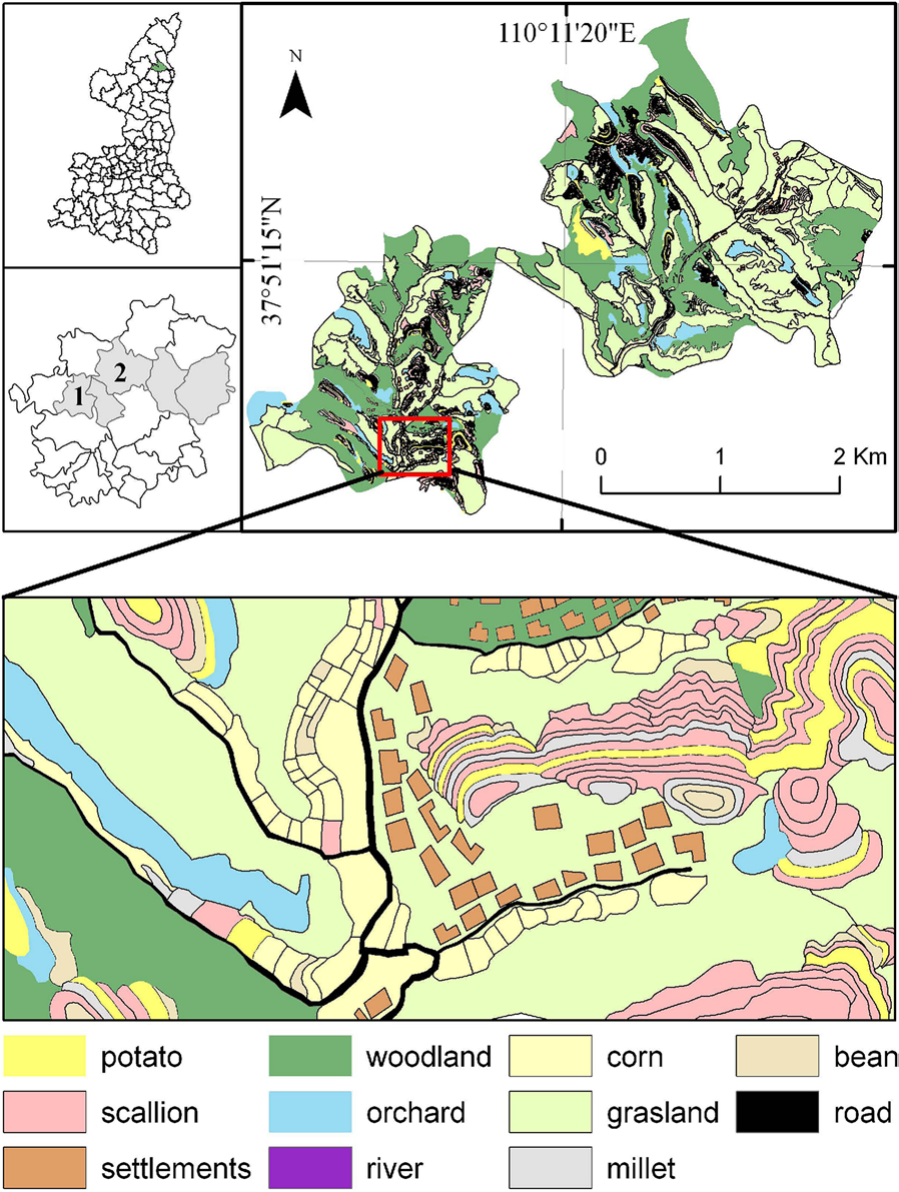
Land-use types in the sample villages of the Gaoqu Commune, Mizhi County, Shaanxi Province, 2009

Local farmers in Gaoqu have a tradition of planting cash crops, particularly potatoes and scallions. However, during the construction of the surrounding potato base in areas around Shaanxi Province, such as Inner Mongolia and Gansu Province, the land area dedicated to potatoes dwindled in the Gaoqu Commune. Statistics showed that the area dedicated to potato cropping decreased by 169.4 ha from 2006 to 2009, whereas the area dedicated to scallion farming increased by 150.4 ha. Five villages (shown in Fig. 1) occupied 60 % of the total increased area. Of these, Matiwa and Jiangxingzhuang were selected as the sample villages. The changes in planting area dedicated to the two cash crops are the outcomes of changes in farmer land-use behavior.

Land use in these two sampled villages was classified into eleven types: potato cropland, scallion cropland, residential land (for settlements), woodland, orchard, river, corn cropland, grassland, millet cropland, bean cropland and road (Fig. 1). The Gaoqu Commune has been a member of the third Batch Demonstrated Commune (an honorary title conferred by the government) of Shaanxi Province since 2010. To promote scallion planting, the local government in Gaoqu subsequently began implementing the “12th Five Year Plan,” which includes three improvements compared with previous measures: (1) the government’s responsibility to respect farmers’ willingness to implement land-use policies was made explicit; (2) specific assistance measures, such as techniques of breeding, planting and pest control, provided by the government were made explicit; (3) government supervision and management of the behavior of scallion farmer cooperatives were made explicit and include the methods by which purchasing contracts are negotiated between farmers and the scallion farmer cooperative. Thus, the changes in local government measures, emergence of future standardization of scallion farmer cooperatives, and influence of the GFG project and market coupled with heterogeneous farmer households provides a dynamic platform for analyzing the formation of farmer land-use behaviors and further clarifying the mechanisms of interaction between farmers and agricultural policy.

### Data sources

This survey was supported by the local government, and no specific permissions were required for the survey locations. The sample villages were selected with the help of advice from the local government and statistical data on the changes in area used for cash crops. The changes in potato and scallion planting areas from 2006 to 2009 in our two sampling villages are shown in Table 1. The percentage of potato planting area in Mativa and Jiangxingzhuang decreased dramatically by 63 and 38 %, respectively, whereas the scallion planting area increased by 282 and 156 %, respectively.

**Table 1:** Change in potato and scallion area in two sample villages (ha). *Data source*: authors’ survey from 2007 to 2009

Year	Village Matiwa		Village Jiangxingzhuang	
	Potato	Scallion	Potato	Scallion
2006	20	7.8	23.3	11.6
2009	7.4	29.8	14.3	29.7

The primary data collection in the study area was conducted from 2007 to 2009 (Fig. 1), and the field studies did not involve endangered or protected species. The surveys were performed according to the farmer households’ willingness, and anonymity was maintained, with the participants’ personal information only used for research and their information kept confidential. A participant could refuse to answer any question at any time. This study and consent procedures were approved by the Ethics Committee of Northwest University of China. Most of the farmer households preferred verbal answers to the survey questions as opposed to writing out their responses. Therefore, we simply wrote their answers in the questionnaires ourselves. The questionnaire was divided into three sections: (1) farmers’ personal and family characteristics, indicators such as household size, income, consumption, education status and livelihood division between agricultural and non-agricultural activities were included; (2) farmers’ planting status, indicators such as total cultivated land area and each type area, the input and output of each crop land types; (3) farmers’ awareness of market risk, the effect of policy and strategy of crop rotation. In fact, there are many factors influencing farmers’ awareness of market risk. To simplify the issue, we assume that if the farmer household knows the price change trend of crop *k*, this implies that there is no market risk for the household in the planting of crop *k*; otherwise, the household will face market risk in this paper. Using social survey methods, the demographic and socio-economic characteristics of all the households in these two villages (the number of Household Registration is 200) were collected. Only 96 farmer households who spend most of the time working at home were investigated directly because of the fixed survey time (between July and August annual). With the help of village cadres, the information of the other farmer households was surveyed by the phone. A valid sample of 152 households spanning the 3 years with an overall response rate of 76 % was obtained. A handheld Global Positioning System (GPS) device was used to identify farm location/land ownership for the surveyed farmers.

Two different policy levels are included in the model. One is the GFG project starting from 1999, and the other is represented by local agriculture development measures. The GFG policy is designed to convert cropland to grassland and woodland. Local agriculture measures represent the main policies influencing farmer households’ land-use behavior on cropland. To illustrate the influence of local measures on farmers’ land-use behavior, we designed three scenarios.

- Scenario I: The local government focuses primarily on controlling pests and diseases and not on the supervision and management of the scallion farmer cooperatives. The relationship between scallion farmer cooperatives and farmers is loose. No purchasing contracts are in place among them. The market risk is assumed by the farmers.
- Scenario II: The local government focuses on controlling pests and diseases and also on the supervision and management of scallion farmer cooperatives. The relationship between the scallion farmer cooperatives and farmers is close, and purchasing contracts are in place. Both the farmers and scallion farmer cooperatives assume a level of market risk.
- Scenario III: The scallion farmer cooperatives organize the purchasing contract and control pests and diseases. The market risk is assumed by the scallion farmer cooperatives.

### Methodology

#### Conceptual framework of farmer household decision making

**Fig. 2:**
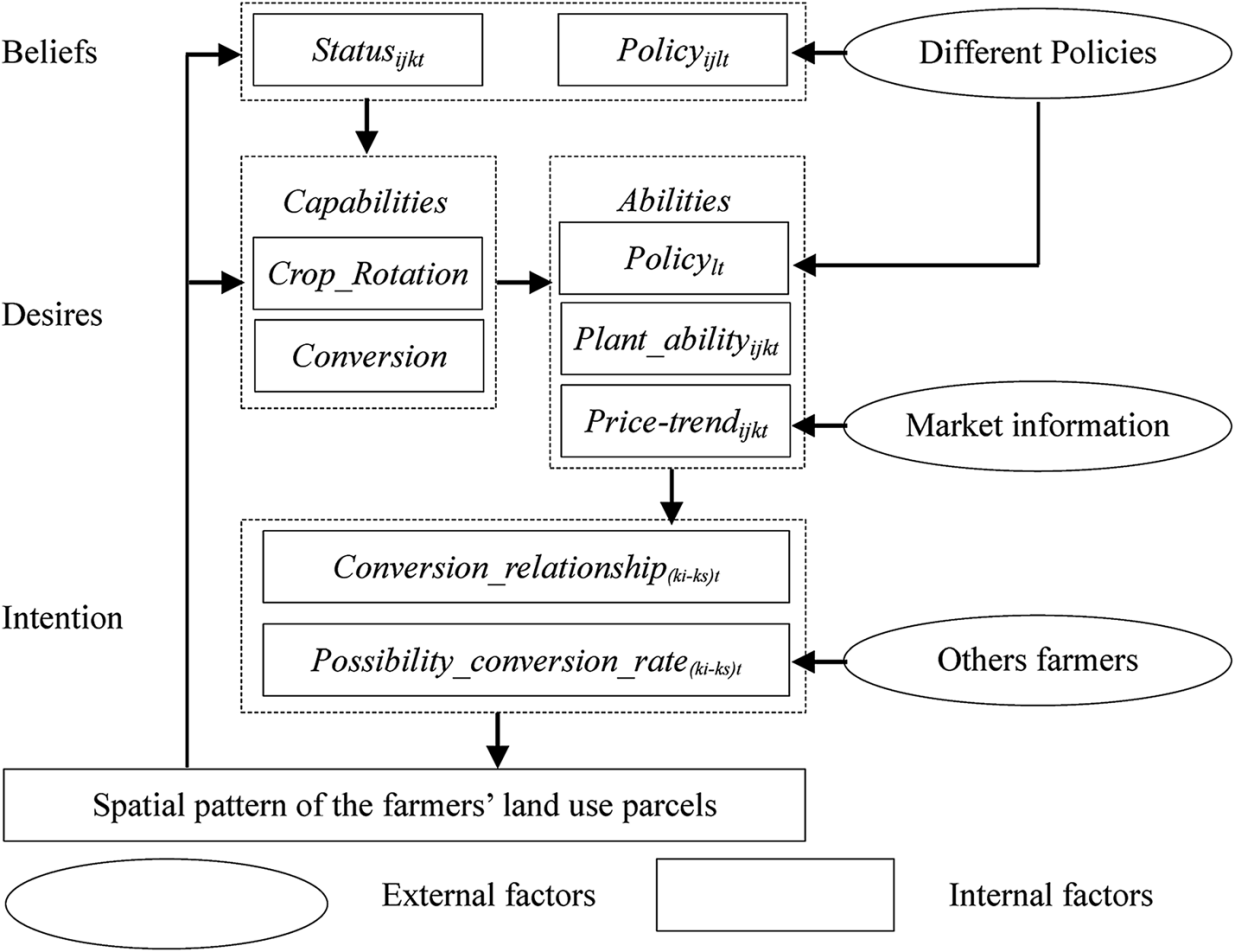
Conceptual framework of farmer household decision-making and its spatial interactions

In our study, the decision makers are farmer households or groups that have a common interest in land use. The conceptual framework of the decision making in farmer household is depicted in Fig. 2. Farmer households or groups are described as having the following characteristics:

1. Farmer households are assumed to have access to information on only a limited part of their environment and themselves but have the ability to learn. They can communicate with each other and learn from the same or different farmer groups as well as the environment;
2. Farmer households are capable of reasoning, communicating and negotiating policies and deciding whether to violate policies if they are unfavorable to their intentions.

The conceptual framework that we propose (Fig. 2) is an extended version of the classic BDI architecture with the addition of two new components: farmer household *Capabilities* and *Abilities*. *Capabilities* are abstract plans of actions that farmers can use to act upon their environment. In our study, *Capabilities* include a *Crop_Rotation* plan and *Conversion* plan. The former refers to the maintenance of the current state of crops, whereas the latter refers to converting the current state of planted crops according to the potential benefits of planting different crops.

Farmer *Abilities* refers to the ability of farmers to convert abstract plans to specified plans. The *abilities* parameter is essential for turning the abstract plan into the specified plan. First, the farmer household will judge whether the policy has an effect on their land use, and whether to take part in this policy. Then, the farmer households adopt different plans for different policies. According to the scope of the policy’s effect, there are two policy types, the national policy and the local measures. In this area, the national policy is GFG policy. However, the GFG policy has no effects on the farmer household behavior as long as the lands are not enrolled in the GFG policy. Therefore, we will pay more attention to the effect of the local measures on the farmer households’ behavior. As for the land not taken part in the GFG policy, the conversion direction will be determined by the local measures.

Through farmer *Abilities*, the *Capabilities* of farmers are turned into specified plans, namely, the desires that the farmer households would like to see implemented. Farmer households are usually capable of interacting with other farmers to gain insight into their actions. Combined with a farmer household’s social and economic status, the intention of the farmer households will be formed. By executing the specified intention, a new spatial pattern of farmer households’ land use will emerge, and the spatial pattern will have an effect on the farmer households’ beliefs and capabilities in the future.

#### Description of the farmer household decision-making model

The decision-making model used in the study is expressed in Eq. (1). Three variables are included in the model, *B*_*ijkt*_, *D*_*ijkt*_ and *I*_*ijkt*_, which refer to the beliefs, desires and intentions of land-use type *k* of farmer *i* in farmer group *j* at time *t*, respectively. Namely, the action of farmers at time *t* + 1 is affected by the BDI of farmers at time *t* according to:

1 $$A_{ijkt + 1} = \{ B_{ijkt} ,D_{ijkt} ,I_{ijkt} \}$$

The three main variables expressed in Eq. (1) are elaborated below.

1. Farmer’s beliefs

The term belief refers to the information that the farmer has on his/her current environment. A farmer’s beliefs are affected by policies at various levels (such as national policies or local measures), market information on crop prices, and current state of his/her land use. Thus, three variables are included in the farmer’s beliefs: *Policy*_*ijlt*_, *Price_trend*_*ijkt*_, and *Status*_*ijkt*_. *Policy*_*ijlt*_ represents the knowing of policy *l* by farmer *i* in farmer group *j* at time *t*, *Price_trend*_*ijkt*_ represents whether farmer *i* in farmer group *j* at time *t* knows the product price change trend of land-use type *k* over the last 3 years, and *Status*_*ijkt*_ represents the status of land-use type *k* of farmer *i* in farmer group *j* at time *t*, meaning the area and location of the different land use of farmer *i* owned.

The differences between the three scenarios mentioned in the section of data sources are related to the parties responsible for pest and disease control, with the local government usually supplying the control techniques through regular training. However, the scallion farmer cooperatives often offer face-to-face field training. Obviously, the efficiency of the latter is higher, and it is more easily accepted by farmers. A simple analysis of the effects of teaching techniques assumes that if the technique is taught by the scallion farmer cooperatives, then the entire farmer group can master the technique. If the training is supplied by the government, farmers whose agriculture practices are generally limited to subsistence farming cannot address their production problems. In addition, to further simplify the analysis, we assume that if there were purchasing contracts between scallion farmer cooperatives and farmers before 2010, these contracts will extend to 2015.

The parameter *Price_trend*_*ijkt*_ is used to represent the market risk. If the farmer household knows the price change trend of crop *k*, this implies that there is no market risk for the household in the planting of crop *k*; otherwise, the household will face market risk.

The crop planting status represents the current environmental information under certain policy and market situations. Equation (2) is designed to provide information on the crop planting status:

2 $$Status_{ijkt} = Area_{ijkt} \times Income_{ijkt} /\sum\limits_{k = 1}^{n} {(Area_{ijkt} \times Income_{ijkt} )}$$

In the equation, *Status*_*ijkt*_ represents the importance of land-use type *k* to farmer *i* in farmer group *j* at time *t* (Eq. 2). Larger *Status*_*ijkt*_ values for land-use type *k* indicate the greater importance of *k* to the farmer. *Area*_*ijkt*_ represents the area of land-use type *k* for farmer *i* in farmer group *j* at time *t*, and *Income*_*ijkt*_ represents the income from land-use type *k* for farmer *i* in farmer group *j* at time *t*. *n* represents the land use types of farmer *i* owned. The order of importance among the crops represents information obtained for the current crop planting.

(2) Farmer’s desires

The *abilities* parameter is essential for turning the abstract plan into the specified plan. First, the farmer household will judge whether the policy has an effect on their land use, and whether to take part in this policy. Then, the farmer households adopt different plans for different policies. According to the scope of the policy’s effect, there are two policy types, the national policy and the local measures. In this area, the national policy is GFG policy. However, the GFG policy has no effects on the farmer household behavior as long as the lands are not enrolled in the GFG policy. Therefore, we will pay more attention to the effect of the local measures on the farmer households’ behavior. As for the land not taken part in the GFG policy, the conversion direction will be determined by the local measures. The conversion direction refers to whether a farmer household will (or will not) maintain the crop planting status. The conversion direction is affected by the farmer household’s *policy_type, plant_ability* and *price* of the relevant crops. Therefore, there are 3 variables included in *Abilities* related by the following (Eq. 3):

3 $$ability_{ijkt} = policy_{lt} \times plant\_ability_{ijkt} \times price\_trend_{ijkt}$$

In this equation, *ability*_*ijkt*_ represents the possibility of turning the abstract plan into the specified plan by farmer *i* in farmer group *j* at time *t* for land-use type *k*, with a value of 1 indicating that the abstract plan can be turned into the specified plan and a value of 0 indicating that it cannot; *policy*_*lt*_ represents the influence of policy *l* at time *t*; *plant_ability*_*ijlt*_ represents the planting ability of farmer *i* in farmer group *j* at time *t* under different scenarios *l*; and *price_trend*_*ijkt*_ is the same as mentioned above.

(3) Farmer’s intentions

The term intention refers to the farmers’ committed plans. The intention of a farmer household will be influenced by other farmers. In this paper, the main effect of the other farmers is on the conversion quantity. The conversion quantity refers to the amount of area that will be converted. The conversion quantity is influenced by the *Possibility_conversion_rate* and *Conversion_relationship* among the different conversion crops. Therefore, the farmer household’s intention can be expressed as Eq. (4):

4 $$Intention_{ijkt} = Ability_{ijkt} \times Possiblity\_conversion\_rate_{(ki - ks)t} \times Conversion\_relationship_{(ki - ks)t}$$

where *Possibility_conversion_rate*_*(ki*-*ks)t*_ represents the average conversion possibility from cash crop *k*_*i*_ to *k*_*s*_ at time *t* and *Conversion_relationship*_*(ki*-*ks)t*_ represents the quantity relationship of conversion between cash crop *k*_*i*_ to *k*_*s*_ at time *t.*

According to the actual conditions of the study area, the critical rule depends on the profit per hectare for different cash crops. If the profit per hectare of *k*_*i*_ in Farmer group I is more than that of *k*_*s*_ in Farmer group II, a conversion from *k*_*s*_ to *k*_*i*_ would occur. Otherwise, a conversion from *k*_*i*_ to *k*_*s*_ would occur. *Possibility_conversion_rate*_*(ki*-*ks)t*_ is expressed in Eq. (5):

5 $$Possibility\_conversion\_rate_{{(ki - k_{s} ) {\text{t}}}} = \frac{1}{n}\left( {\sum\limits_{i = 1}^{n} {Area_{{ijk_{s} t}} /\sum\limits_{i = 1}^{\text{n}} {\sum\limits_{k = 1}^{m} {Area_{ijkt} } } } } \right)$$

where *Area*_*ijkst*_ and *Area*_*ijkt*_ represent the areas of converted crops and all crops, respectively, of farmer *i* in farmer group *j*; *n* represents the number of farmers in farmer group *j*, and *m* represents the number of crop types in farmer group *j*. This modelling pattern is a result of assuming that (1) farmer land-use behavior is based on bounded rationality instead of maximum benefit and (2) the standard represents the average trend of land-use structure by farmers.

The results of the *Conversion_relationship*_*(ki*-*ks)t*_ among the different conversion crops are dependent on the actual crop production in the study area. Based on our surveys, scallions require 3 years from planting to harvest. The first transplantation area is approximately three times that of the breeding area (maximum time is 4 years, and minimum is 2 years). The percent of the area of the second transplantation to that of the first is 1. Once 1 hectare of cropland is converted to scallion land, the farmer will have 7 hectares of scallion land after 3 years. Therefore, the conversion quantity of scallion is 7 times the area of other cropland.

## Results

### Classification of farmer households

Based on the cluster analysis of the interview data, the farmer households were classified and farmers were aggregated into groups based on age, education, average cropping area and crop planting profit. A hierarchical cluster analysis was performed using the average linkage between group selections.

The characteristics of each group are summarized in Table 2. The first group is referred to as ‘scallion-planting farmers,’ and it contains approximately 10 % of all farmers with scallion as their main cash crop. These farmers are relatively young and well educated, and most started planting scallions in 2004. The second group is referred to as ‘potato-planting farmers,’ and it contains approximately 21 % of all farmers with potato as their main cash crop. Compared to the first group, this group is relatively older and less well educated. The third group contains approximately 27 % of all farmers and has an average age of 55. Although potato is their main cash crop, this group is older than the second group. The fourth group is referred to as older farmers, and it contains approximately 42 % of all farmers and has an average age of 60. The agriculture practices of this group are generally limited to subsistence farming, and a rotation plan is their primary land-use mode. The first group and second group acquired a larger cropping area by leasing land from the fourth group.

**Table 2:** Classification of the farmer groups. *Data source*: authors’ survey in 2007, 2008 and 2009

Farmer group	Farmer number	Average age	Education	The average land area per farmer (ha)	Dominant cash crop
Farmer group I	16	42	Senior high school	1.8	Scallion
Farmer group II	30	47	Junior high school	2	Potato
Farmer group III	41	55	Elementary school	1.5	Potato
Farmer group IV	65	60	Elementary school	0.3	–

### Analysis of farmer beliefs

In the study area, farmers generally plant orchard trees, potatoes or corn. Scallion was first planted in 2000. The land-use status as calculated with Eq. 2 is shown in Table 3. Due to the number of farmers is larger, the farmer listed in Table 3 were typical farmers in each farmer groups.

**Table 3:** The importance of land-use type for different farmer groups. *Data source*: authors’ survey in 2007, 2008 and 2009

Farmer		Scallion	Potato	Orchard	Corn
Farmer groups	Farmers				
Farmer group I	1	70	4	17	9
	2	76	4	14	6
	3	90	10	0	0
Farmer group II	4	0	48	51	1
	5	0	60	36	4
	6	0	43	45	12
Farmer group III	7	0	54	42	4
	8	0	79	9	12
	9	0	62	34	4
Farmer group IV	10	0	0	0	100
	11	0	87	0	14

The order of importance of land-use types varied greatly among farmer groups. This variability reflects the diversity of the farmer households’ livelihoods. For Farmer group I, the crops (ordered in descending importance) are scallion, orchard, potatoes and corn; for group II, the crops are orchard, potato and corn; for group II, the crops are potatoes, orchard and corn for group III, and for group IV, the crops are corn and potatoes. Although the market risk of planting scallions in the study area was still uncertain in 2004, Farmer group I began to plant scallions, which is now considered the most important crop in the group’s cropping practices. Farmer group II chose orchard as its most important crop because of its lower market risk than scallions, and having greater profit than other crops except scallions. Farmer group III regarded potatoes as its most important crop in its market-declined condition. The differences in the groups’ choices for their most important crop could be a result of their ability to know market risk. Therefore, the ability to know market risk among different farmer groups (in descending order) is group I, group II, group III and group IV.

Some research have revealed that crop diversification and livelihood diversity can reduce the market risk, and embody the bounded rationality of farmer households’ decision-making (Chen et al. 2012a; Li et al. 2013). Crop diversification is an important livelihood strategy among farmers in the study area. To avoid market risk, Farmer group I retains a certain number of parcels to plant other crops although planting scallion yields the most profit, and this practice is similar to that of other farmer groups and indicates that the strategy of maximizing benefits is not the optimal choice. Most farmers choose bounded rationality as their land-use strategy because of unclear market information on crop prices and uncertain supporting measures (e.g., supervising and managing the purchasing contracts by the local government).

### Analysis of farmer desire

According to our surveys, we found that the main rotation plans of farmers include potato–bean–potato–millet, millet–potato and millet–millet–bean–bean. Most farmers adopted the ‘potato–bean–potato–millet’ rotation plan in the study area; therefore, we treated this plan as the farmer’s *Crop_Rotation* plan.

In the study area, there are four primary cash crops: scallion, orchard, potato, and corn. Corn is used for livestock feed and orchard trees are planted for at least five years, which means that the farmers cannot cut down these trees for other cash crops. Therefore, we treated the conversion that occurs occurring between scallions and potatoes as the other abstract plan, namely the *Conversion*.

Our surveys indicated that the profit from potatoes is 18,000 yuan/ha, and the profit from scallions is 67,500 yuan/ha. A farmer wanting to adopt the conversion plan would incur 14,400 yuan in opportunity costs according to the designed rule described in Farmers’ Intention. However, the profit from scallions is 27,000 yuan. Therefore, even if the opportunity cost was incurred before the maturation of the scallions, the farmer would still earn 12,600 yuan. This reasoning explains why the conversion cost is not considered in the farmer household decision making model.

In this paper, the GFG policy has no effects on the farmer household behavior as long as the lands in question are not enrolled in the GFG policy. Because of its focus on other land, the farmer household behavior is affected by local measures. Thus, the policy is expressed as follows:

6 $$policy_{lt} = \left\{ {\begin{array}{*{20}l} 1 \hfill & \quad{l = local} \hfill & {measures} \hfill \\ 0 \hfill &\quad {l = GFG} \hfill & {policy} \hfill \\ \end{array} } \right.$$

According to the concept and local measure scenarios, *plant_ability*_*ijlt*_ and *price*_*ijkt*_ can be derived as follows:

7 $$plant\_ability_{ijlt} = \left\{ {\begin{array}{*{20}c} 1 & {i \notin subsistence\,farmer\quad and\quad l = scenarioI\,or\,scenarioII} \\ 0 & \quad\quad\quad{l = GFG\quad policy} \\ \end{array} } \right.$$

8 $$price_{ijkt} = \left\{ {\begin{array}{*{20}c} 1 & \quad{contract = true} \\ 0 & \quad{contract = false} \\ \end{array} } \right.$$

**Fig. 3:**
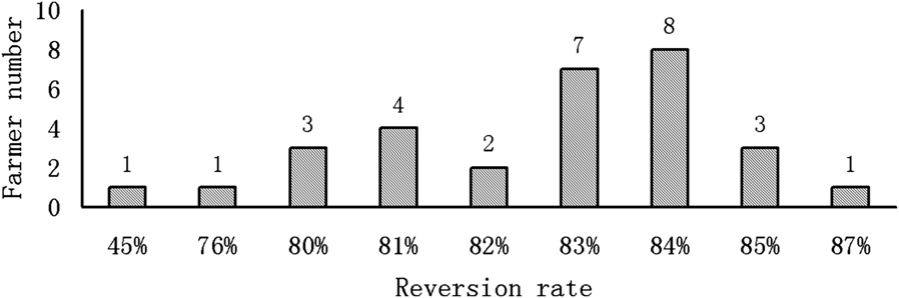
Conversion percent and farmer numbers for Farmer group II in 2009

The local government began helping farmers with the breeding and control of pests and diseases connected with planting scallions in 2006. All of the farmers except those in Farmer group IV have been able to plant scallions since 2006 (*Plant_ability*_*ijlt*_ = 1). According to the interview results, farmers in groups I and II signed contracts with the scallion farmer cooperatives. Accordingly, the value of *price*_*ijkt*_ for Farmer group I and Farmer group II is equal to 1, whereas the value of *price*_*ijkt*_ for the other farmer groups is equal to 0. According to the designed conversion rule, only Farmer group II converted their potatoes to scallions. The other groups adopted the rotation plan. The results of the analysis of farmers’ desires are shown in Table 4 and Fig. 3.

**Table 4:** Errors in the farmer land-use decision-making model’s simulation of different farmer groups. *Data source*: authors’ survey in 2007, 2008 and 2009

Year	Index of error	Farmer group I		Farmer group II		Farmer group III		Farmer group IV		Error area percent of total cropland area of all farmer groups
		Crop-rotation plan	Conversion plan	Crop-rotation plan	Conversion plan	Crop-rotation plan	Conversion plan	Crop-rotation plan	Conversion plan	
2007	Number of farmers	6	0	2	2	3	2	10	12	–
	Error area percent of total cropland area of each farmer group	3.70 %	0	0.70 %	6.80 %	1.40 %	0.70 %	6.50 %	0.50 %	4.70 %
2008	Number of farmers	6	0	2	2	3	2	10	12	–
	Error area percent of total cropland area of each farmer group	3.70 %	0	0.70 %	9.80 %	1.40 %	1.70 %	6.50 %	7.20 %	6.90 %
2009	Number of farmers	6	0	2	2	3	2	10	12	–
	Error area percent of total cropland area of each farmer group	3.70 %	0	0.70 %	4.60 %	1.40 %	3.10 %	6.50 %	4.90 %	6.30 %

The error is the difference between the simulation and real data

For the *Conversion* plan and *Crop_Rotation* plan, we can draw the following conclusions.

- Compared with the actual conversion in 2009, the results of the simulation shows that 95.4 % of the farmers in Farmer group II adopted the *Conversion* plan, which demonstrates that the conversion rule designed in farmer desire section is accurate.
- Certain farmers in groups III and IV also adopted the *Conversion* plan (Table 4). The percent of farmers who adopted the *Conversion* plan in groups III and IV was 5 and 18 %, respectively. These results may reflect a strong desire by these farmers to perform the conversion.

### Analysis of farmer intention

Based on our surveys, the *Possibility_conversion_rate* of the scallion area to the total for each farmer is first calculated. The average percent of conversion is calculated with Eq. (5). The conversion percent is 84 %, and we can draw the following conclusions according to results of Eq. (4) and Eq. (5).

- Compared with the designed conversion percent, the actual average conversion percent for Farmer group II was 83 % (Fig. 3) because of the different sized parcels. Only two farmers had an average conversion percent below 80 %. Although two farmers adopted the *Conversion* plan, they still had concerns related to the market risk of planting scallions, and they preferred to diversify their strategy.
- The percent of the area that adopted the other rotation plan to the total area for Farmer group IV was relatively low (6.5 %, see Table 4), which indicates that the rotation plan is appropriate for most farmers in the study area.

In addition, unlike the *Crop_Rotation* plan, the error percent of the area adopting the *Conversion* plan to the total crop area of different farmer groups is a dynamic rather than stable number (Table 4). For example, the error percent for Farmer group II was 6.8 % in 2007 and changed to 9.8 % in 2008 and 4.6 % in 2009. A similar phenomenon occurred for the other groups as well, and these changes are related to the percent of scallion breeding area to first transplant area. The error percent in 2008 was greater than that in other years because farmers had more opportunity to choose the same acreage parcel in 2008 than they did in 2009.

Overall, the average area error percent from 2007 to 2009 was within 7 % of the area of total cropland in all farmer groups. The accurate percentage of farmers adopting the *Crop_Rotation* plan and *Conversion* plan was over 80 % of the total farmer population (Table 4). Therefore, the designed model and conversion rule are feasible.

### Analysis of three policy scenarios for the conversion to scallion

Planting scallions is more profitable than cultivating other crops in the study area. The local government is devoted to expanding scallion cropland. To promote scallion cropping and determine feasible local policies, we analyzed the effects of three policy scenarios on farmer land-use behavior.

According to the three scenarios described in farmer’s belief section and conversion rule described in farmer’s intension section, we simulated the land-use conversion in 2015 for the study area (Table 5).

**Table 5:** Conversion results of different farmer groups in different scenarios in 2015 (ha)

Scenario types	Farmer group I	Farmer group II	Farmer group III	Farmer group IV	Total
Scenario I	0	0	0	0	0
Scenario II	0	0	27.3	0	27.3
Scenario III	0	0	27.3	15.4	42.7

In policy scenario I, the conversion area of scallion is equal to 0 (Table 5), which indicates that all farmer groups will adopt the *Crop_Rotation* plan in 2015. Although none of the groups will convert their cropland to scallion land, each farmer group has its own reason. Farmer groups I and II have a stronger ability to understand the market risk than other groups, but they adjust the land-use structure to their own satisfaction and are unwilling to convert their other cropland to scallion. Because of the lack of purchasing contracts, Farmer group III assumes the market risk by itself; therefore, the value of *price*_*ijkt*_ for Farmer group III is equal to 0 and the quantity conversion for Farmer group III is also equal to 0. Because of age and labor problems, farmers in group IV have no ability to convert cropland to scallion. In addition, Farmer group IV assumes the market risk by itself, which is similar to Farmer group III. Therefore, the quantity conversion for Farmer group IV is also equal to 0. In policy scenario II, only Farmer group III will convert its cropland to scallion. Compared with the risk in policy scenario I, the market risk faced by Farmer group III is lower in scenario II. With a contract signed between Farmer group III and the scallion farmer cooperative, both will assume the market risk.

Therefore, Farmer group III will convert its cropland to scallion under this scenario. Similar to scenario I, the reasons for declining to convert in Farmer groups I, II and IV remain the same.

In scenario III, Farmer groups III and IV will convert their cropland to scallion, whereas Farmer groups I and II decline to convert for the same reasons as in scenario I. Based on the assumptions designed in farmer’s intension section and assumption of market risk by the scallion farmer cooperatives, farmers in Farmer groups III and IV will be willing to convert their cropland to scallion land under scenario III.

The results of the simulation showed that when the market risk assumed by farmers was low, the area of conversion of conversion increased. Therefore, if the local government wants to promote scallion planting, it should reduce the market risk to farmers, which may be performed by including standardized operations and effectively supervising the activity of scallion farmer cooperatives, especially by helping them improve their ability to anticipate price change trends for main cash crops. Because most of the conversion occurs in Farmer groups III and IV, the government should focus its attention on these groups. If the age and land-area percent of different farmer groups are considered (the land area percent of Farmer groups III and IV were 35 and 11 %, respectively), the government should focus its attention to Farmer group III.

If only the area of conversion is considered, scenario III is the most favorable. However, if the economic tolerance of the scallion farmer cooperatives is considered, the sustainability of scenario III is questionable because of the scallion farmer cooperatives will face high economic pressure. Compared with scenario III, the sustainability of the scallion farmer cooperatives in scenario II is greater. Therefore, the rational choice among the three scenarios is scenario II.

## Conclusions and discussion

Studies that have considered the learning process when discussing human decision making may be divided into two types based on their expression of the learning process: random selection processes, which depend on the actions paying off (Sobel 2000; Satake et al. 2007), and interaction processes, which occur between individuals and groups (Chen et al. 2012b; Fleischman et al. 2014; Chen et al. 2015). In our study, the farmer household decision-making process is the result of farmer characteristics and other farmer actions. This paper constructed a CA-BDI model to analyze and model farmer household land use behavior in Mizhi County of western China’s Shaanxi Province. Our simulation was conducted according to certain parameter values that characterize the learning process in our study and in others (Galef 1992; Chen et al. 2012a).

Two parameters included in the CA-BDI model, namely capability and ability, were designed to capture the bounded rational decision-making process, and the interaction among different farmers was analyzed to reveal the communication among farmer households. In order to capture the diversity in farmers’ capabilities and abilities, three scenarios were designed, and farmer households’ respond were simulated. Based on the simulations, the diversity of the farmers’ decision-making process needed to be considered when different local economic measures were implemented. The simulation result showed that the CA-BDI decision-making framework designed in this paper can express the formation of farmer households’ decision-making, and explored how the changes in decision making can affect land use changes through the interaction among different farmer households.

The scenario simulation and the CA-BDI model presented in this paper offer several advantages. First, scenario simulation is increasingly recognized as a useful tool for exploring changes in social ecological systems and helps shape the future or adapt to changing conditions (Rounsevell et al. 2005; Brady et al. 2012). Local agriculture measures were given more attention in this paper. To reveal the effect of local measures on farmers’ land use behavior, three scenarios were designed. Similar to other studies (Vincent 2007; Chen et al. 2012a), the effect of local measures on the farmers’ behavior was analyzed, which provided the prerequisite for simulating the farmers’ bounded rational behavior and formed the basis for exploring the effects of different local measures. Second, reproducing the farmers’ bounded rational decision-making process and understanding how it will change has drawn increasing attention (Quang et al. 2008). Most studies using the BDI decision-making structure have adopted the theory of maximum benefit to reveal the mechanism of change in farmers’ land use behavior (Balke and Gilbert 2014; Chen et al. 2015). After being given the effect of local agriculture measures and analyzing the interaction among farmers, farmers might learn from other farmers, and they might change their land use behavior. Compared to the decision-making framework based on the theory of maximum benefit, CBDI better reflected the actual farmers’ land use behavior.

In addition to the possible advantages offered by the approaches, there are limitations. First, the scenario setting was relatively simple. For example, the settings for supervision and the management of purchasing contracts were simple in this paper. It was assumed that the effectiveness is achieved as long as supervision and management were conducted by the local government. Although the effects of local agriculture measures were revealed, their evolution and effects on the farmers’ behavior were not included in this paper. This will be the focus of our future work. Second, only the interaction among different farmers was analyzed in this paper. The interaction among farmers included the interaction within a farmer group and the interaction among different farmer groups. These two interaction types could have an effect on farmers’ behavior. The lack of analysis of the interaction among the same farmers influenced the accuracy of farmers’ behavior analysis. Third, the parameter value (*Possibility_conversion_rate*) is objectively determined through surveys and not subjectively assigned. In addition, social networks can be important and effective as enforcement and compliance tools for environmental regulations (Bodin and Crona 2009; Barabasi 1999). Although the interactions between different farmer groups or within the same farmer group have been explored, the impact of social networks on learning has not been well considered. Therefore, determining an approach to reveal the interaction among the same farmers to capture the actual farmers’ land use behavior, and the effects of social networks on farmer household decision making will be our research focus in the future.

Through a case study of Mizhi County, Shaanxi Province, China, the effects of the local measures on farmer households are discussed, and under the designed framework, the factors belief, desire and intention are also discussed. The quantitative relationships among these factors are used to explore the formation and change mechanisms in farmer land-use decision-making and analyze the bounded rational decisions made by farmers and learned behavior that occurs undergoing environmental change. Through a comparison of three policy scenarios, policy recommendations for both local government and farmer groups are provided. To promote scallion planting, we propose that the local government help scallion farmer cooperatives understand the market risk and standardize their operations and supervise contracts between scallion cooperatives and farmers.
